# *N*,*N*′-Dimethyl-*N*′′-(trichloroacet­yl)phospho­ramide

**DOI:** 10.1107/S1600536813030389

**Published:** 2013-11-09

**Authors:** Vladimir Ovchynnikov

**Affiliations:** a Department of Inorganic Chemistry, Kiev National Taras Shevchenko University, Vladimirskaya St. 64/13, Kiev 01601, Ukraine

## Abstract

In the title compound, C_4_H_9_Cl_3_N_3_O_2_P or CCl_3_C(O)NHP(O)(NHCH_3_)_2_, the P atom has a strongly distorted tetra­hedral geometry due to the formation of intermolecular strong hydrogen bonds involving the N atoms. In the crystal, N—H⋯O=P and N—H⋯O=C hydrogen bonds connect the mol­ecules into a two-dimensional array parallel to (100). An intra­molecular P⋯O contact [P⋯O = 2.975 (3) Å] is observed. The CCl_3_ group is rotationally disordered, with occupancies of 0.60 (3) and 0.40 (3)

## Related literature

For the use of carbacyl­amido­phosphates as potential new ligands for metal ions, see: Skopenko *et al.* (2004 ▶); Znovjyak *et al.* (2009 ▶); Yizhak *et al.* (2013 ▶); Gubina *et al.* (2009 ▶). For their biological activity, see: Amirkhanov *et al.* (1996 ▶); Rebrova *et al.* (1984 ▶). For P=O and C=O bond lengths, see: Mizrahi & Modro (1982 ▶); Amirkhanov *et al.* (1997 ▶); Gubina & Amirkhanov (2000 ▶). For the preparation of tri­chloro­acetyl­amido­phospho­ric acid dichloranhydride, see: Kirsanov & Derkach (1956 ▶).
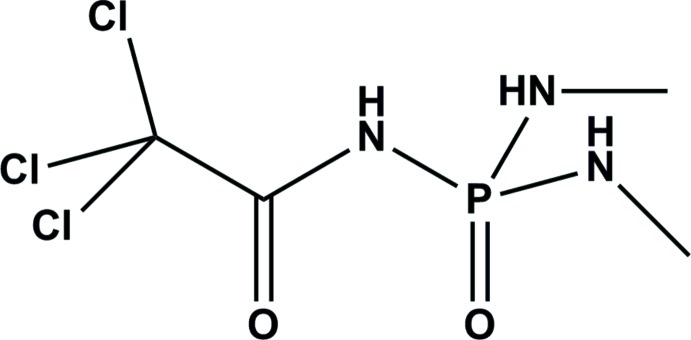


## Experimental

### 

#### Crystal data

C_4_H_9_Cl_3_N_3_O_2_P
*M* *_r_* = 268.46
Monoclinic, 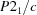

*a* = 10.231 (2) Å
*b* = 8.754 (2) Å
*c* = 12.826 (3) Å
β = 101.27 (3)°
*V* = 1126.6 (4) Å^3^
*Z* = 4
Mo *K*α radiation
μ = 0.93 mm^−1^
*T* = 293 K
0.4 × 0.3 × 0.3 mm

#### Data collection

Enraf–Nonius CAD-4 diffractometer
3806 measured reflections
1908 independent reflections
1419 reflections with *I* > 2σ(*I*)
*R*_int_ = 0.060
3 standard reflections every 200 reflections intensity decay: 1%

#### Refinement

*R*[*F* ^2^ > 2σ(*F* ^2^)] = 0.077
*wR*(*F* ^2^) = 0.195
*S* = 1.08
1908 reflections
157 parameters
33 restraints
H atoms treated by a mixture of independent and constrained refinement
Δρ_max_ = 0.82 e Å^−3^
Δρ_min_ = −0.77 e Å^−3^

### 

Data collection: *CAD-4 EXPRESS* (Enraf–Nonius, 1995 ▶); cell refinement: *CAD-4 EXPRESS*; data reduction: *XCAD4* (Harms & Wocadlo, 1996 ▶); program(s) used to solve structure: *SHELXS97* (Sheldrick, 2008 ▶); program(s) used to refine structure: *SHELXL97* (Sheldrick, 2008 ▶); molecular graphics: *ORTEP-3 for Windows* (Farrugia, 2012 ▶); software used to prepare material for publication: *WinGX* (Farrugia, 2012 ▶).

## Supplementary Material

Crystal structure: contains datablock(s) I. DOI: 10.1107/S1600536813030389/bg2520sup1.cif

Structure factors: contains datablock(s) I. DOI: 10.1107/S1600536813030389/bg2520Isup2.hkl

Supplementary material file. DOI: 10.1107/S1600536813030389/bg2520Isup3.cml

Additional supplementary materials:  crystallographic information; 3D view; checkCIF report

## Figures and Tables

**Table 1:** Hydrogen-bond geometry (Å, °)

*D*—H⋯*A*	*D*—H	H⋯*A*	*D*⋯*A*	*D*—H⋯*A*
N1—H1⋯O1^i^	0.81 (3)	2.00 (4)	2.782 (5)	164 (5)
N3—H3⋯O1^i^	0.81 (3)	2.21 (4)	2.953 (4)	153 (4)
N2—H2⋯O2^ii^	0.81 (3)	2.38 (4)	3.077 (5)	146 (6)

Symmetry codes: (i) 
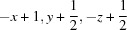
; (ii) 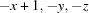.

**Fig. 1.**
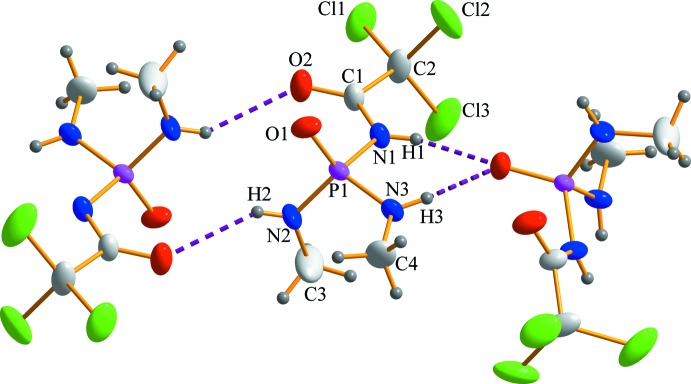
A view of the title compound (I) showing the atom-numbering scheme and the formation of three type of hydrogen bonds (dashed lines). Displacement ellipsoids drawn at a 30% probability level.

**Fig. 2.**
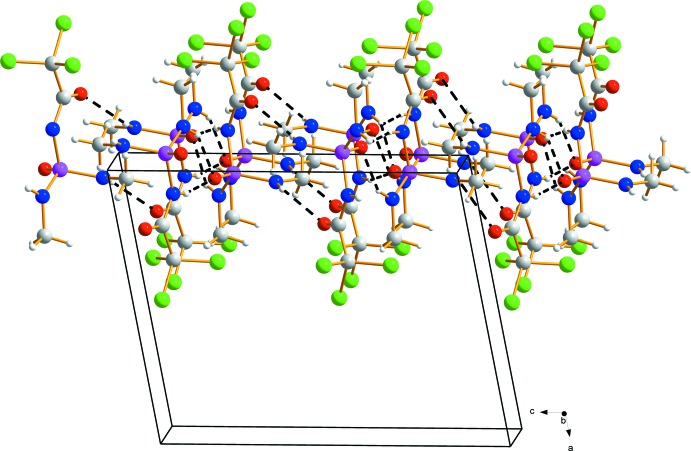
Packing view of (I) along the b axis. Only the major fraction of the CCl3 group has been represented.

## supplementary crystallographic information

### 1. Introduction

Carbacyl­amido­phosphates of the general formula RC(O)NHP(O)R'_2_ are potential new ligands for metal ions (Skopenko *et al.*, 2004; Znovjyak *et al.*, 2009; Gubina *et al.*, 2009). Many of these compounds also show biological activity (Amirkhanov *et al.*, 1996, Rebrova *et al.*, 1984). This work reports the structure of N,N'-Di­methyl-N''-trichloracetyl­phospho­ramide (C_4_H_9_N_3_O_2_PCl_3_) (I).

### 2.1. Synthesis and crystallization

The dichloranhydride of tri­chloro­acetyl­amido­phospho­ric acid was prepared according to the method reported by Kirsanov (Kirsanov & Derkach, 1956). The dioxane solution (200 ml) of dichloranhydride of tri­chloro­acetyl­amido­phospho­ric acid (27.9 g, 0.1 mol) was placed in a three-neck round-bottomed flask and cooled by ice to 268 K. Then the dry methyl­amine was bubbled through the dioxane solution of CCl_3_C(O)NHP(O)Cl_2_ under stirring until the solution became alkaline. The temperature was not allowed to rise above 278 K. The stirring was continued for 1 h and the solution was left under ambient conditions. H_2_NCH_3_·HCl was filtered off after 12 h and the filtrate was evaporated. The oily precipitate of I was added to acetone which led to the formation of a white crystalline powder (yield 80%). White crystals suitable for X-ray analysis were obtained from slow evaporation of a 2-propanol solution.

### 2.2. Refinement

H atoms of methyl groups were placed at calculated positions and treated as riding on the parent atoms, with U_iso_(H) = 1.5 U_eq_(C). H atoms of the amide group were located in a difference Fourier map and and further refined with similarity restraints for d(N—H) and U_iso_(H) = 1.2U_eq_(N). The CCl_3_ group apperas rotationally disordered around the C1—C2 bond, with occupations of 0.60/0.40 (3)

### 3. Results and discussion

In the title compound (I), the phospho­rus environment has a strong distorted tetra­hedral conformation due to the formation of strong N1—H1···O1 and N3—H3···O1 hydrogen bonds (Table 2, Fig.1). The N1—P—N3 angle has a value 98.72° and as a consequence there is an increase in the O1—P1—N3 and O1—P1—N1 angles (119.2° and 111.29°, respectively). The orientation of the C(O) and P(O) groups differs from the conformation of most CAF-ligands (Gubina & Amirkhanov, 2000), the angle between the O2C1N1 and N1PO1 planes having a value 57.3° (the pseudo-torsion angle O=C···P=O is -53.39°).

In the crystal, two inter­molecular N–H···O=P hydrogen bonds connect molecules into a chain and a third N—H···O=C hydrogen bond connects the chains into a 2D array parallel to (100) (Fig.2). An intra­molecular P···O contact is also present in the crystal [d(P···O) = 2.975 (3) Å], sorter than the sum of commonly accepted Van der Waals Radii (3.3 Å).

### Crystal data

**Table:**

C_4_H_9_Cl_3_N_3_O_2_P	*F*(000) = 544
*M**_r_* = 268.46	*D*_x_ = 1.583 Mg m^−^^3^
Monoclinic, *P*2_1_/*c*	Mo *K*α radiation, λ = 0.71073 Å
Hall symbol: -P 2ybc	Cell parameters from 2348 reflections
*a* = 10.231 (2) Å	θ = 2.0–27.1°
*b* = 8.754 (2) Å	µ = 0.93 mm^−^^1^
*c* = 12.826 (3) Å	*T* = 293 K
β = 101.27 (3)°	Block, colorless
*V* = 1126.6 (4) Å^3^	0.4 × 0.3 × 0.3 mm
*Z* = 4	

### Data collection

**Table:**

Enraf–Nonius CAD-4 diffractometer	*R*_int_ = 0.060
Radiation source: fine-focus sealed tube	θ_max_ = 25.0°, θ_min_ = 2.0°
Graphite monochromator	*h* = −12→12
ω/Θ scans	*k* = 0→10
3806 measured reflections	*l* = −15→15
1908 independent reflections	3 standard reflections every 200 reflections
1419 reflections with *I* > 2σ(*I*)	intensity decay: 1%

### Refinement

**Table:**

Refinement on *F*^2^	Primary atom site location: structure-invariant direct methods
Least-squares matrix: full	Secondary atom site location: difference Fourier map
*R*[*F*^2^ > 2σ(*F*^2^)] = 0.077	Hydrogen site location: inferred from neighbouring sites
*wR*(*F*^2^) = 0.195	H atoms treated by a mixture of independent and constrained refinement
*S* = 1.08	*w* = 1/[σ^2^(*F*_o_^2^) + (0.1369*P*)^2^] where *P* = (*F*_o_^2^ + 2*F*_c_^2^)/3
1908 reflections	(Δ/σ)_max_ < 0.001
157 parameters	Δρ_max_ = 0.82 e Å^−^^3^
33 restraints	Δρ_min_ = −0.77 e Å^−^^3^

### Special details

**Table:**

Geometry. All esds (except the esd in the dihedral angle between two l.s. planes) are estimated using the full covariance matrix. The cell esds are taken into account individually in the estimation of esds in distances, angles and torsion angles; correlations between esds in cell parameters are only used when they are defined by crystal symmetry. An approximate (isotropic) treatment of cell esds is used for estimating esds involving l.s. planes.

### Fractional atomic coordinates and isotropic or equivalent isotropic displacement parameters (Å^2^)

**Table:**

	*x*	*y*	*z*	*U*_iso_*/*U*_eq_	Occ. (<1)
P1	0.55195 (8)	0.17846 (12)	0.16971 (7)	0.0379 (4)	
Cl1	0.0184 (6)	0.1759 (10)	0.0571 (8)	0.098 (2)	0.60 (3)
Cl2	0.1528 (7)	0.2717 (16)	0.2675 (4)	0.108 (2)	0.60 (3)
Cl3	0.1588 (6)	0.4570 (7)	0.0816 (9)	0.114 (3)	0.60 (3)
Cl1A	0.0175 (8)	0.1557 (17)	0.0770 (15)	0.108 (5)	0.40 (3)
Cl2A	0.1466 (9)	0.321 (3)	0.2554 (10)	0.134 (5)	0.40 (3)
Cl3A	0.1524 (15)	0.434 (2)	0.050 (2)	0.185 (9)	0.40 (3)
O1	0.5607 (3)	0.0273 (4)	0.2191 (3)	0.0597 (8)	
O2	0.2813 (3)	0.0649 (4)	0.0732 (3)	0.0683 (10)	
N1	0.3994 (3)	0.2573 (4)	0.1646 (3)	0.0461 (8)	
H1	0.396 (5)	0.335 (5)	0.197 (4)	0.055*	
N2	0.5807 (5)	0.1646 (5)	0.0515 (3)	0.0668 (12)	
H2	0.602 (6)	0.080 (5)	0.036 (5)	0.080*	
N3	0.6418 (3)	0.3153 (4)	0.2290 (3)	0.0495 (9)	
H3	0.605 (4)	0.373 (6)	0.263 (3)	0.059*	
C1	0.2872 (4)	0.1851 (5)	0.1188 (3)	0.0508 (10)	
C2	0.1579 (4)	0.2697 (6)	0.1287 (4)	0.0724 (15)	
C3	0.5757 (12)	0.2923 (9)	−0.0197 (5)	0.130 (4)	
H3A	0.6401	0.2781	−0.0641	0.195*	
H3B	0.5954	0.3845	0.0208	0.195*	
H3C	0.4882	0.2994	−0.0632	0.195*	
C4	0.7862 (4)	0.3127 (8)	0.2458 (5)	0.0814 (17)	
H4A	0.8171	0.4026	0.2152	0.122*	
H4B	0.8145	0.2236	0.2126	0.122*	
H4C	0.8226	0.3102	0.3207	0.122*	

### Atomic displacement parameters (Å^2^)

**Table:**

	*U*^11^	*U*^22^	*U*^33^	*U*^12^	*U*^13^	*U*^23^
P1	0.0356 (5)	0.0399 (6)	0.0419 (6)	0.0050 (3)	0.0166 (4)	0.0026 (4)
Cl1	0.049 (3)	0.089 (3)	0.137 (4)	−0.0087 (18)	−0.028 (3)	−0.040 (3)
Cl2	0.069 (3)	0.172 (6)	0.090 (3)	0.021 (3)	0.0296 (16)	−0.044 (4)
Cl3	0.059 (2)	0.058 (2)	0.198 (5)	0.0179 (14)	−0.041 (3)	−0.004 (3)
Cl1A	0.037 (3)	0.109 (7)	0.176 (9)	0.002 (3)	0.017 (4)	−0.075 (6)
Cl2A	0.040 (3)	0.161 (9)	0.208 (11)	−0.018 (4)	0.043 (4)	−0.130 (8)
Cl3A	0.116 (7)	0.096 (8)	0.292 (17)	0.032 (5)	−0.084 (9)	0.022 (9)
O1	0.0463 (14)	0.0482 (19)	0.086 (2)	0.0032 (12)	0.0153 (13)	0.0251 (16)
O2	0.0524 (17)	0.062 (2)	0.087 (2)	0.0047 (14)	0.0037 (15)	−0.0299 (18)
N1	0.0347 (15)	0.053 (2)	0.0496 (17)	0.0054 (14)	0.0070 (12)	−0.0148 (16)
N2	0.103 (3)	0.050 (3)	0.060 (2)	0.004 (2)	0.049 (2)	−0.0094 (19)
N3	0.0321 (16)	0.061 (2)	0.059 (2)	−0.0007 (13)	0.0171 (13)	−0.0123 (17)
C1	0.045 (2)	0.051 (3)	0.055 (2)	0.0054 (16)	0.0051 (17)	−0.0146 (19)
C2	0.039 (2)	0.072 (4)	0.099 (4)	0.006 (2)	−0.005 (2)	−0.034 (3)
C3	0.263 (11)	0.083 (5)	0.062 (3)	0.001 (6)	0.076 (5)	0.006 (3)
C4	0.038 (2)	0.090 (4)	0.119 (5)	−0.008 (2)	0.023 (2)	−0.016 (3)

### Geometric parameters (Å, º)

**Table:**

P1—O1	1.462 (3)	N1—H1	0.81 (3)
P1—N2	1.605 (4)	N2—C3	1.437 (8)
P1—N3	1.608 (4)	N2—H2	0.81 (3)
P1—N1	1.696 (3)	N3—C4	1.451 (5)
Cl1—C2	1.744 (6)	N3—H3	0.81 (3)
Cl2—C2	1.791 (7)	C1—C2	1.544 (6)
Cl3—C2	1.748 (7)	C3—H3A	0.9600
Cl1A—C2	1.768 (8)	C3—H3B	0.9600
Cl2A—C2	1.710 (9)	C3—H3C	0.9600
Cl3A—C2	1.753 (9)	C4—H4A	0.9600
O2—C1	1.199 (5)	C4—H4B	0.9600
N1—C1	1.342 (5)	C4—H4C	0.9600
			
O1—P1—N2	109.5 (2)	C1—C2—Cl3A	106.1 (7)
O1—P1—N3	119.3 (2)	Cl2A—C2—Cl3A	109.4 (7)
N2—P1—N3	108.0 (2)	Cl1—C2—Cl3A	98.8 (8)
O1—P1—N1	111.30 (18)	C1—C2—Cl1A	110.2 (4)
N2—P1—N1	109.4 (2)	Cl2A—C2—Cl1A	107.6 (5)
N3—P1—N1	98.72 (18)	Cl3—C2—Cl1A	117.3 (7)
C1—N1—P1	121.8 (3)	Cl3A—C2—Cl1A	108.4 (6)
C1—N1—H1	120 (3)	C1—C2—Cl2	106.2 (4)
P1—N1—H1	118 (3)	Cl1—C2—Cl2	110.4 (5)
C3—N2—P1	123.4 (4)	Cl3—C2—Cl2	109.7 (5)
C3—N2—H2	122 (4)	Cl3A—C2—Cl2	124.1 (9)
P1—N2—H2	114 (4)	Cl1A—C2—Cl2	101.5 (6)
C4—N3—P1	122.0 (4)	N2—C3—H3A	109.5
C4—N3—H3	120 (3)	N2—C3—H3B	109.5
P1—N3—H3	116 (4)	H3A—C3—H3B	109.5
O2—C1—N1	125.7 (4)	N2—C3—H3C	109.5
O2—C1—C2	119.9 (4)	H3A—C3—H3C	109.5
N1—C1—C2	114.3 (4)	H3B—C3—H3C	109.5
C1—C2—Cl2A	115.0 (6)	N3—C4—H4A	109.5
C1—C2—Cl1	110.9 (4)	N3—C4—H4B	109.5
Cl2A—C2—Cl1	115.0 (5)	H4A—C4—H4B	109.5
C1—C2—Cl3	111.0 (4)	N3—C4—H4C	109.5
Cl2A—C2—Cl3	95.1 (8)	H4A—C4—H4C	109.5
Cl1—C2—Cl3	108.6 (5)	H4B—C4—H4C	109.5

### Hydrogen-bond geometry (Å, º)

**Table:**

*D*—H···*A*	*D*—H	H···*A*	*D*···*A*	*D*—H···*A*
N1—H1···O1^i^	0.81 (3)	2.00 (4)	2.782 (5)	164 (5)
N3—H3···O1^i^	0.81 (3)	2.21 (4)	2.953 (4)	153 (4)
N2—H2···O2^ii^	0.81 (3)	2.38 (4)	3.077 (5)	146 (6)

Symmetry codes: (i) −*x*+1, *y*+1/2, −*z*+1/2; (ii) −*x*+1, −*y*, −*z*.
